# Childhood deprivations predict late-life cognitive impairment among older adults in India

**DOI:** 10.1038/s41598-022-16652-y

**Published:** 2022-07-27

**Authors:** T. Muhammad, Paramita Debnath, Shobhit Srivastava, T. V. Sekher

**Affiliations:** 1 Department of Family & Generations, International Institute for Population Sciences, Mumbai, Maharashtra 400088 India; 2 International Institute for Population Sciences, Mumbai, 400088 India; 3 Department of Survey Research & Data Analytics, International Institute for Population Sciences, Mumbai, India

**Keywords:** Geriatrics, Public health

## Abstract

Large population-based studies on the associations of childhood factors with late-life cognition are lacking in many low and middle income countries including India. In this study, we assessed the prevalence of late-life cognitive impairment and examined the associations of childhood socioeconomic status (SES) and health conditions with cognitive impairment among older adults in India. Data for this study were derived from the Longitudinal Ageing Study in India conducted in 2017–18. The effective sample size was 31,464 older adults aged 60 years and above. Cognitive functioning was measured through five global domains (memory, orientation, arithmetic function, executive function, and object naming). The overall score ranged between 0 and 43, and the score was reversed indicating cognitive impairment. Descriptive statistics along with mean scores of cognitive impairment were presented. Additionally, moderated multivariable linear regression models were employed to examine the association between explanatory variables, including childhood SES and health conditions and late-life cognitive impairment. The mean score of cognitive functioning among the study participants was 21.72 (CI 2.64–21.80). About 15% of older adults had poor health conditions, and 44% had lower financial status during their childhood. Older adults who had a fair health during their childhood were more likely to suffer from cognitive impairment in comparison to older adults who had good health during their childhood (Coef: 0.60; CI 0.39, 0.81). In comparison to older adults who had good childhood financial status, those who had poor childhood financial status were more likely to suffer from cognitive impairment (Coef: 0.81; CI 0.56, 1.07). Older adults who had fair childhood health status and poor childhood financial status were more likely to suffer from cognitive impairment in comparison to older adults who had good childhood health and good financial status (Coef: 1.26; CI 0.86, 1.66). Social policies such as improving educational and financial resources in disadvantaged communities and socioeconomically poor children and their families, would help to enhance a better cognitive ageing and a healthy and dignified life in old age.

## Introduction

The growing number of older adults around the world poses serious challenges related to health, and older adults without any social security benefits, savings, and affordability of good quality care are the ones to suffer the most^1^. The risk of cognitive impairment is expected to be the most prominent with increasing age, and to a great extent, determined by childhood cognitive function^2–4^. However, studies differ substantially in identifying the factors responsible for faster cognitive decline with age^5^. Studies commonly identified childhood socioeconomic status as a representative of childhood adversities, while the association they found were inconsistent; for instance, a study found higher childhood socioeconomic status showing with the slower global cognitive decline with increasing age, whereas others found a faster cognitive decline^6,7^.

In recent years, the concentration of cognitive impairment cases such as dementia among older adults is rising^8–10^. However, the burden differs between subgroups in communities across countries based on abilities that can be categorized into memory, conceptualization, attention, language, knowledge, and spatial ability, each one of which is influenced by both biological as well as environmental factors^11^. Although the mechanisms have not been explicated, investigators have hypothesized that chronic diseases, functional disabilities, poor health behaviours and decreased social interactions may be potential mediators of childhood deprivations resulting in poor mental health in late-life^12–14^. Several longitudinal and interventional studies have reported the beneficial effects of non-pharmacological interventions such as resistance, aerobic and balance exercises on cognitive functions of older adults^15,16^. Furthermore, cognitive decline is also associated with numerous other factors such as age, gender, marital status, level of education, and overall health conditions in the past^17^. Some studies explained how marital status and a higher level of education motivated individuals to maintain a healthier lifestyle; that stimulates brain activities in day-to-day lives, and community participation/social involvement during the absence of a partner/spouse might be associated with better mental wellbeing^18–20^.

India is home to millions of children who are exposed to deprivation in terms of basic health, nutrition that facilitate survival, growth and development^21^. The poor state of children in India can be reflected by the increase in the cases of stunting, wasting and malnutrition in recent years^22^. Further, children who were being more exposed to early life deprivation were also associated with the development of lower cognitive reserve for the brain that might lead to poor learning experience and higher functional illiteracy^23^. Childhood with socio-economic disadvantage might also lead to pathways towards clinical and social risks such as substance use, social isolation and mental distress, which might worsen late-life cognitive function^24^. According to brain reserve hypothesis, environment plays an important role that influences brain plasticity and thus affects the intelligence, education and occupation that determine the cognitive functioning^25,26^. However, some studies argued that cognitive decline in older age is not determined by single life-period, rather due to exposure to reserve-related factors^27^, and the accumulation of multiple traumas throughout the life course^28^.

Since early life factors for late-onset diseases have been well documented in the epidemiological field^29,30^, understanding the independent association of early life factors with late-life cognition may be more beneficial in designing cognition-enhancing interventions which may be equally effective regardless of individuals’ genetic susceptibility^27^. Although it has received empirical attention in several developed countries^31–33^, and some developing countries like China^11,34,35^, South Africa^36^, and Indonesia^37^, large population-based studies on the associations of childhood factors with late-life cognition are lacking in many low and middle income countries including India. In this study, we tested whether childhood deprivation is associated with cognitive impairment in older age, using data from the survey on older adults with comprehensive information on their socioeconomic and health status. In the analysis, we focused on markers of childhood deprivation, including childhood health conditions and early life family financial status of older Indian adults.

## Methods

### Data

**Figure 1:**
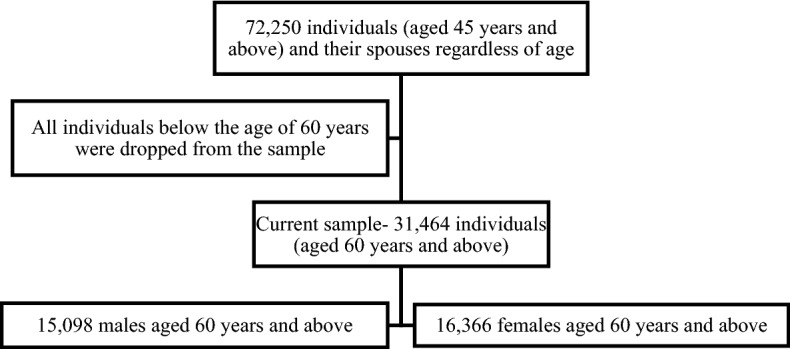
Flowchart for the study sample selection.

Data for this study were derived from the recent release of the Longitudinal Ageing Study in India (LASI) wave 1. The LASI is a full-scale national survey of scientific investigation of the health, economic, and social determinants and consequences of population ageing in India, conducted in 2017–18 by the International Institute for Population Sciences (IIPS) in partnership with national and international institutions^38^. The LASI is a nationally representative survey of 72,250 individuals aged 45 and above across all states and union territories of India. The main objective of the survey is to study the health status and the social and economic well-being of older adults in India. LASI adopted a multistage stratified area probability cluster sampling design to arrive at the eventual units of observation: older adults age 45 and above and their spouses irrespective of age. The survey adopted a three-stage sampling design in rural areas and a four-stage sampling design in urban areas. In each state/Union Territory, the first stage involved the selection of Primary Sampling Units (PSUs), that is, sub-districts (Tehsils/Talukas), and the second stage involved the selection of villages in rural areas and wards in urban areas in the selected PSUs. In rural areas, households were selected from selected villages in the third stage. However, sampling in urban areas involved an additional stage. Specifically, in the third stage, one Census Enumeration Block (CEB) was randomly selected in each urban area. In the fourth stage, households were selected from this CEB. The socio-demographic and health-related information of respondents in the LASI survey was assessed using the face-to-face interviews which were conducted using computer-assisted personal interview (CAPI). The detailed methodology, with the complete information on the survey design and data collection, was published in the survey report^38^. The present study is conducted on eligible respondents aged 60 years and above. The total sample size for the present study is 31,464 (15,098 males and 16,366 females) elderly persons aged 60 years and above. Figure 1 represents the flowchart for the study sample selection. All methods were performed in accordance with the relevant guidelines and regulations.

### Variable description

#### Outcome variable

Cognitive functioning was assessed using continuous measures of five global domains of cognition (memory, orientation, arithmetic function, executive function, and object naming), adapted from the Mini-Mental State Examination (MMSE)^39^, and the cognitive module of the Health and Retirement Study, the China Health and Retirement Longitudinal Study (CHARLS), and the Mexican Health and Aging Study (MHAS)^40,41^. Memory was measured using immediate word recall and delayed word recall. Orientation was measured using time and place measures. The arithmetic function was measured through backward counting, a serial seven subtraction task and a task involving two computations^38,40^. Additionally, paper folding (folding a piece of paper according to instructions), pentagon drawing (drawing intersecting circles) and object naming methods were followed to measure the cognitive functions among older adults^41^ (Cronbach’s alpha: 0.70). The overall score of composite index of cognitive function ranged between 0 and 43, and a higher score indicated better cognitive functioning. Further, for the analytical purpose, the score was reversed to assess the cognitive impairment among older adults and thus after reversing, the higher score indicated higher levels of cognitive impairment. In our study, the respondents who received assistance during the cognition module were excluded from the analysis^38^.

#### Key explanatory variables

The main explanatory variables were childhood health status (good, fair and poor) and childhood SES (good, average, poor). In the LASI survey, childhood health condition was measured using the question “Now I want to ask you about your overall childhood health up to age 16. In general, would you say your childhood health was very good, good, fair, poor, or very poor based on what you remember, or what you heard or perceived from your parents?” The responses were coded as good if he/she reported “very good and good” and fair if he/she reported “fair” and poor if he/she reported “poor and very poor”^42^. Additionally, financial status of the respondents’ family during childhood (childhood SES) was measured through the question, “Now, think about your family when you were growing up, from birth to age 16. Compared to other families in your community, would you say your family during that time was pretty well off financially, about average, or poor?” The response was coded as good if they reported “pretty well off financially”, average if reported “average” and poor if reported “poor”.

#### Mediating factors

As per the above-mentioned literature, the following factors which were shown to potentially mediate the associations between childhood factors and late-life cognition were included in the current analysis. Social participation was measured through the question- “Are you a member of any of the organizations, religious groups, clubs, or societies?” and the response was coded as no and yes. Physical activity was categorized as frequent (every day), rare (more than once a week, once a week, one to three times in a month), and never. The question through which physical activity was assessed was “How often do you take part in sports or vigorous activities, such as running or jogging, swimming, going to a health centre or gym, cycling, or digging with a spade or shovel, heavy lifting, chopping, farm work, fast bicycling, cycling with loads?”^43^. If the older adult was ill-treated in the last 1 year, then it was coded as “yes”; otherwise “no”.

The probable major depression among older adults with symptoms of dysphoria, was calculated using the CIDI-SF (Short Form Composite International Diagnostic Interview) with a score of 3 or more indicating “diagnosed with depression”^44^. The Cronbach’s alpha value for the CIDI-SF scale was 0.668. This scale estimates a probable psychiatric diagnosis of major depression and has been validated in field settings and widely used in population-based health surveys^38^. Self-rated health was coded as good which includes excellent, very good, and good whereas poor includes fair and poor^45^. Difficulty in activities of daily living (ADL) was coded as no and yes. The Cronbach’s alpha value for ADL scale was 0.869. ADL is a term used to refer to normal daily self-care activities (such as movement in bed, changing position from sitting to standing, feeding, bathing, dressing, grooming, personal hygiene, etc.). Difficulty in instrumental ADL (IADL) was coded as no and yes^46^. The Cronbach’s alpha value for IADL scale was 0.879. These include activities that are not necessarily related to the fundamental functioning of a person, but they let an individual live independently in a community. Morbidity was coded as no morbidity, 1 and 2 + ^47^. The variable morbidity was created using the data on chronic diseases which include hypertension, chronic heart diseases, stroke, any chronic lung disease, diabetes, cancer or malignant tumor, any bone/joint disease, neurological/psychiatric disease, or high cholesterol^47^.

#### Control variables

Several socio-demographic variables were controlled in the analysis. They include, age, which was recoded as young old (60–69 years), old-old (70–79 years), and oldest-old (80 + years); sex, which was recoded as male and female; educational status (equivalent to the International Standard Classification of Education (ISCED) categories)^48^, which was recoded as no education/primary not completed, primary (ISCED 1), secondary (ISCED 2, 3 and 4) and higher; working status, which was recoded as never worked, currently working, currently not working and retired; marital status, which was categorized as currently married, widowed, and others (divorced/separated/never married); and living arrangement, which was categorized as living alone, living with a spouse, living with spouse and children, and living with others.

Further, the monthly per capita consumption expenditure (MPCE) quintile was assessed using household consumption data. Sets of 11 and 29 questions on the expenditures on food and non-food items, respectively, were used to canvas the sample households. Food expenditure was collected based on a reference period of seven days, and non-food expenditure was collected based on reference periods of 30 days and 365 days. Food and non-food expenditures have been standardized to the 30-day reference period. The MPCE is computed and used as the summary measure of consumption^38^. The variable was divided into five quintiles i.e., from poorest to richest. Religion was coded as Hindu, Muslim, Christian, and Others. Caste was recoded as Scheduled Tribe (ST), Scheduled Caste (SC), Other Backward Classes (OBCs), and others. The ST refers to a large number of aboriginal ethnic groups or the indigenous population in the country. The SC includes the population that is socially segregated and financially/economically weak by their low status as per the Hindu caste hierarchy. The STs and SCs are among the most disadvantaged and discriminated socio-economic groups as per Government of India official classification. The OBC is the group of people who were identified as “educationally, economically and socially backwards”. The OBCs are considered low in the traditional caste hierarchy but are higher in status than the STs/SCs. The “other” caste category is identified as those who are having higher social status, mostly belonging to the upper caste Hindus^49^. The place of residence was coded as rural and urban. The regions of India were coded as North, Central, East, Northeast, West, and South.

### Statistical analysis

Descriptive statistics along with mean (95% confidence interval) was presented in the study. Additionally, moderated multiple linear regression analysis^50^ was used to examine the association between the outcome variable (cognitive impairment) with other explanatory variables. The estimates were presented in the form of adjusted coefficients calculated at 95% confidence interval (CI). Additionally, standard beta coefficients were presented in the results. The regression diagnostics for heteroscedasticity^51^, multicollinearity^52^, and outliers were performed via computation of variance inflation factors (VIFs) and visual inspection of residual plots for the regression models. The complex survey design effects were adjusted by using STATA *svyset* and *svy* commands. The whole statistical analyses were performed by using Stata version 14^53^. Model-1 provides the estimates adjusted for all the mediating and control variables considered in the study. Model-2 represents the adjusted estimates of interaction effects of childhood health (good fair, and poor) and childhood financial status (good, average, poor) with cognitive impairment among older adults. Model-3 represents the estimates from the stratified analysis (categorical results) of childhood health and childhood financial status.

### Ethics approval and consent to participate

The Indian Council of Medical Research (ICMR), Delhi and Institutional Review boards (IRBs) of all partner institutions extended the necessary guidance and ethical approval for conducting the LASI survey. The partner institutions included IIPS, Mumbai; Harvard T.H. Chan School of Public Health (HSPH), Boston; University of Southern California (USC), Los Angeles; ICMR-National AIDS Research Institute (NARI), Pune; and the Regional Geriatric Centres (RGCs), ministry of health and family welfare (MoHFW). Informed consent was obtained from all subjects and/or their legal guardian(s) in accordance with human subject protection protocols.

## Results

Table 1 represents the socioeconomic profile of the older adults in India. It was found that about 3.7% of the older adults had poor health conditions in their childhood. Nearly 43.9% of the older adults had a poor childhood SES. Around 68.0% of the older adults had no education or their primary education was incomplete. Nearly 26.4% of the older adults were currently not working. About 36.2% of older adults were widowed, whereas about 61.6% were currently married. Nearly 5.7% of older adults lived alone, and about 20.3% lived with their spouses. About 95.5% of older adults had no social participation. Nearly 69.3% of the older adults reported that they have never done any physical activity. Almost 5.2% of the older adults were ill-treated in last 1 year. About 8.7% of the older adults were suffering from depression. Nearly 48.6% of the older adults reported having poor self-rated health. About 23.8% and 48.3% of the older adults reported having difficulty in ADL and IADL, respectively. About 23.9% of the older adults had 2 + chronic conditions.

**Table 1:** Socio-economic profile of older adults in India, (n = 31,464).

Background factors	Sample	Percentage
**Childhood deprivation factors**		
**Childhood health status**		
Good	27,227	86.5
Fair	3077	9.8
Poor	1160	3.7
**Childhood financial status**		
Good	2633	8.4
Average	15,009	47.7
Poor	13,822	43.9
**Individual factors**		
**Age**		
Young–old	18,410	58.5
Old–old	9501	30.2
Oldest–old	3553	11.3
**Sex**		
Male	14,931	47.5
Female	16,533	52.6
**Education**		
No education/primary not completed	21,382	68.0
Primary	3520	11.2
Secondary	4371	13.9
Higher	2191	7.0
**Working status**		
Never worked	8315	26.4
Currently working	9397	29.9
Not currently working	11,470	36.5
Retired	2282	7.3
**Marital status**		
Currently married	19,391	61.6
Widowed	11,389	36.2
Others	684	2.2
**Living arrangement**		
Living alone	1787	5.7
Living with spouse	6397	20.3
Living with children and spouse	21,475	68.3
Living with others	1805	5.7
**Social participation**		
No	30,053	95.5
Yes	1411	4.5
**Physical activity**		
Frequent	5651	18.0
Rarely	4023	12.8
Never	21,790	69.3
Ill-treated in last one year*		
Yes	1587	5.2
No	28,840	94.8
**Health-related factors**		
Depression*		
No	27,995	91.3
Yes	2657	8.7
Self-rated health*		
Good	15,850	51.4
Poor	14,961	48.6
Difficulty in ADL*		
No	23,887	76.2
Yes	7449	23.8
Difficulty in IADL*		
No	16,188	51.7
Yes	15,148	48.3
**Chronic disease**		
0	14,773	47.0
1	9171	29.2
2 +	7520	23.9
**Household/community factors**		
**MPCE quintile**		
Poorest	6830	21.7
Poorer	6831	21.7
Middle	6590	21.0
Richer	6038	19.2
Richest	5175	16.5
**Religion**		
Hindu	25,871	82.2
Muslim	3548	11.3
Christian	900	2.9
Others	1145	3.6
**Caste**		
Scheduled caste	5949	18.9
Scheduled tribe	2556	8.1
Other backward class	14,231	45.2
Others	8728	27.7
**Place of residence**		
Rural	22,196	70.6
Urban	9268	29.5
**Region**		
North	3960	12.6
Central	6593	21.0
East	7439	23.6
Northeast	935	3.0
West	5401	17.2
South	7136	22.7
**Total**	31,464	100.0

*Sample size may differ due to missing cases; MPCE, Monthly per capita consumption expenditure; ADL, Activities of daily living; IADL, Instrumental activities of daily living; Percentages are weighted.

Table 2 represents the mean score of cognitive impairment by background characteristics. It was found that the mean score of cognitive impairment was higher among older adults whose childhood status was fair (mean: 22.71; CI 22.46, 22.96). The mean score of cognitive impairment was higher among older adults who had poor financial status of family during their childhood (mean: 23.29; CI 23.16, 23.41). The mean score of cognitive impairment was higher among older adults who had no education (mean: 24.56; CI 24.47, 24.65). Older adults who never worked had higher mean score of cognitive impairment (mean: 22.91; CI 22.75, 23.10). The mean score of cognitive impairment was reported to be higher among older adults who were widowed (mean: 23.88; CI 23.73, 24.02). Older adults who were living with others had higher mean score of cognitive impairment (mean: 24.30; CI 23.93, 24.67). The mean score of cognitive impairment was higher among older adults who had no social participation (mean: 21.88; CI 21.8, 21.97). Older adults who never did physical activity had higher mean score of cognitive impairment (mean: 22.21; CI 22.11, 22.31). The mean score of cognitive impairment was higher among older adults who were ill-treated in last one year (mean: 22.48; CI 22.06, 22.89). Older adults who had depression had a higher mean score of cognitive impairment (mean: 22.85; CI 22.53, 23.16). The mean score of cognitive impairment was higher among older adults who reported to have poor self-rated health (mean: 22.66; CI 22.54, 22.78) and those who had difficulty in ADL and IADL (mean: 23.73; CI 23.54, 23.93) (mean: 23.70; CI 23.57, 23.82).

**Table 2:** Mean score of cognitive impairment among older adults by background characteristics, (n = 24,625).

Background factors	Mean	CI (95% CI)
**Childhood deprivation factors**		
**Childhood health status**		
Good	21.60	21.51–21.69
Fair	22.71	22.46–22.96
Poor	22.01	21.32–22.69
**Childhood financial status**		
Good	19.40	19.13–19.68
Average	20.78	20.67–20.89
Poor	23.29	23.16–23.41
**Individual factors**		
**Age**		
Young-old	20.79	20.69–20.89
Old-old	22.70	22.54–22.85
Oldest-old	24.79	24.49–25.09
**Sex**		
Male	19.59	19.49–19.70
Female	23.88	23.76–23.99
**Education**		
No education/primary not completed	24.56	24.47–24.65
Primary	18.72	18.55–18.89
Secondary	16.40	16.27–16.54
Higher	14.46	14.28–14.63
**Working status**		
Never worked	22.91	22.75–23.10
Currently working	21.00	20.85–21.13
Not currently working	22.79	22.64–22.92
Retired	16.39	16.16–16.60
**Marital status**		
Currently married	20.61	20.51–20.71
Widowed	23.88	23.73–24.02
Others	21.98	21.45–22.51
**Living arrangement**		
Living alone	23.51	23.13–23.89
Living with spouse	21.34	21.16–21.52
Living with children and spouse	21.51	21.42–21.61
Living with others	24.30	23.93–24.67
**Social participation**		
No	21.88	21.80–21.97
Yes	18.72	18.44–19.00
**Physical activity**		
Frequent	20.40	20.22–20.58
Rarely	21.22	21.01–21.43
Never	22.21	22.11–22.31
**Ill-treated in last one year**		
Yes	22.48	22.06–22.89
No	21.69	21.60–21.77
**Health-related factors**		
**Depression**		
No	21.62	21.54–21.71
Yes	22.85	22.53–23.16
**Self-rated health**		
Good	20.89	20.00.78–21
Poor	22.66	22.54–22.78
**Difficulty in ADL**		
No	21.17	21.08–21.26
Yes	23.73	23.54–23.93
**Difficulty in IADL**		
No	20.08	19.97–20.18
Yes	23.70	23.57–23.82
**Chronic disease**		
0	22.20	22.08–22.33
1	21.70	21.55–21.84
2 +	20.86	20.70–21.02
**Household/community factors**		
**MPCE quintile**		
Poorest	23.21	23.03–23.40
Poorer	22.45	22.27–22.63
Middle	21.49	21.31–21.66
Richer	21.12	20.95–21.30
Richest	20.05	19.87–20.23
**Religion**		
Hindu	21.65	21.55–21.74
Muslim	22.30	22.07–22.53
Christian	21.31	21.02–21.59
Others	22.13	21.76–22.49
**Caste**		
Scheduled Caste	23.32	23.13–23.52
Scheduled Tribe	24.24	24.03–24.45
Other Backward Class	21.46	21.33–21.59
Others	20.54	20.40–20.69
**Place of residence**		
Rural	22.88	22.78–22.98
Urban	19.07	18.94–19.20
**Region**		
North	22.11	21.92–22.29
Central	22.04	21.82–22.25
East	22.07	21.87–22.27
Northeast	21.07	20.80–21.33
West	22.03	21.80–22.27
South	20.66	20.50–20.82
**Total**	21.72	21.64–21.80

CI, Confidence interval; ADL, Activities of daily living; IADL, Instrumental activities of daily living; MPCE, Monthly per capita consumption expenditure; p value is based on chi-square test.

Table 3 depicts the regression estimates of cognitive impairment among older adults by their background characteristics. Older adults who had a fair health during their childhood were more likely to suffer from cognitive impairment in reference to older adults who had good health during their childhood (Coef: 0.60; CI 0.39, 0.81). In comparison to older adults who had good childhood financial status, those who had poor childhood financial status were more likely to suffer from cognitive impairment (Coef: 0.81; CI 0.56, 1.07). The likelihood of cognitive impairment was found to be higher among the older adults who had no education in reference to older adults whose education status was higher (Coef: 6.99; CI 6.71, 7.26). Older adults who were widowed were more likely to suffer from cognitive impairment as compared to their married counterparts (Coef: 0.73; CI 0.56, 0.91). Older adults who were currently working were more likely to suffer from cognitive impairment as compared to older adults who never worked (Coef: 0.43; CI 0.22, 0.64). The likelihood of cognitive impairment was higher among older adults who had no social participation in comparison to those who had social participation (Coef: 0.69; CI 0.43, 0.94). Older adults who never involved in physical activity were more likely to suffer from cognitive impairment in reference to older adults who frequently involved in physical activity (Coef: 0.91; CI 0.73, 1.09). The likelihood of cognitive impairment was higher among older adults who had poor self-rated health in comparison to those who had good self-rated health (Coef: 0.72; CI 0.59, 0.86). Older adults who had difficulty in ADL and IADL were more likely to suffer from cognitive impairment in reference to older adults who had no difficulty in ADL and IADL (Coef: 0.66; CI 0.48, 0.84) (Coef: 0.98; CI 0.83, 1.12).

**Table 3:** Regression estimates of cognitive impairment among older adults by their background characteristics, (n = 24,625).

Background factors	Model-1		Model-2		Model-3	
	aCoef. (95% CI)	Beta	aCoef. (95% CI)	Beta	aCoef. (95% CI)	Beta
**Childhood deprivation factors**						
**Childhood health status**						
Good	Ref		Ref			
Fair	0.60* (0.39, 0.81)	0.027	0.59 (− 0.42, 1.59)	0.026		
Poor	− 0.29 (− 0.80, 0.22)	− 0.005	0.75 (− 1.36, 2.86)	0.014		
**Childhood financial status**						
Good	Ref		Ref			
Average	0.11 (− 0.14, 0.35)	0.008	0.1 (− 0.15, 0.35)	0.01		
Poor	0.81* (0.56, 1.07)	0.059	0.84* (0.58, 1.11)	0.06		
**Childhood health status # Childhood financial status**						
Fair # Average			0.14 (− 0.91, 1.18)	0.00		
Fair # Poor			− 0.17 (− 1.23, 0.89)	0.00		
Poor # Average			− 1.08 (− 3.34, 1.19)	− 0.01		
Poor # Poor			− 1.13 (− 3.35, 1.09)	− 0.02		
**Childhood health status and childhood financial status**						
Good and good					Ref	
Good and average					0.10 (− 0.15, 0.35)	0.008
Good and poor					0.84* (0.58, 1.11)	0.060
Fair and good					0.59 (− 0.42, 1.59)	0.006
Fair and average					0.83* (0.48, 1.18)	0.029
Fair and poor					1.26* (0.86, 1.66)	0.036
Poor and good					0.75 (− 1.36, 2.86)	0.003
Poor and average					− 0.23 (− 1.07, 0.62)	− 0.003
Poor and poor					0.46 (− 0.26, 1.18)	0.006
**Individual factors**						
**Age**						
Young–old	Ref		Ref		Ref	
Old–old	1.04* (0.89, 1.19)	0.070	1.04* (0.89, 1.19)	0.07	1.04* (0.89, 1.19)	0.070
Oldest–old	2.65* (2.41, 2.90)	0.111	2.65* (2.41, 2.9)	0.11	2.65* (2.41, 2.90)	0.111
**Sex**						
Male	Ref		Ref		Ref	
Female	1.67* (1.50, 1.84)	0.125	1.67* (1.5, 1.84)	0.12	1.67* (1.50, 1.84)	0.125
**Education**						
No education/primary not completed	6.99* (6.71, 7.26)	0.509	6.99* (6.71, 7.26)	0.51	6.99* (6.71, 7.26)	0.509
Primary	2.75* (2.45, 3.04)	0.138	2.75* (2.45, 3.04)	0.14	2.75* (2.45, 3.04)	0.138
Secondary	1.22* (0.95, 1.49)	0.068	1.22* (0.95, 1.49)	0.07	1.22* (0.95, 1.49)	0.068
Higher	Ref		Ref		Ref	
**Marital status**						
Currently married	Ref		Ref		Ref	
Widowed	0.73* (0.56, 0.91)	0.051	0.73* (0.56, 0.91)	0.05	0.73* (0.56, 0.91)	0.051
Others	− 0.07 (− 0.52, 0.37)	− 0.002	− 0.08 (− 0.52, 0.36)	0.00	− 0.07 (− 0.52, 0.37)	− 0.002
**Living arrangement**						
Living alone	Ref		Ref		Ref	
Living with spouse	0.20 (− 0.16, 0.55)	0.012	0.2 (− 0.16, 0.55)	0.01	0.20 (− 0.16, 0.55)	0.012
Living with children and spouse	− 0.15 (− 0.47, 0.16)	− 0.011	− 0.16 (− 0.47, 0.16)	− 0.01	− 0.15 (− 0.47, 0.16)	− 0.011
Living with others	0.41 (0.01, 0.82)	0.013	0.41 (0, 0.82)	0.01	0.41 (0.01, 0.82)	0.013
**Working status**						
Never worked	0.43* (0.22, 0.64)	0.028	0.43* (0.22, 0.64)	0.03	0.43* (0.22, 0.64)	0.028
Currently working	Ref		Ref		Ref	
Not currently working	0.15 (− 0.03, 0.33)	0.011	0.15 (− 0.03, 0.33)	0.01	0.15 (− 0.03, 0.33)	0.011
Retired	− 0.47* (− 0.73, − 0.21)	− 0.021	− 0.47* (− 0.73, − 0.21)	− 0.02	− 0.47* (− 0.73, − 0.21)	− 0.021
**Social participation**						
No	0.69* (0.43, 0.94)	0.027	0.69* (0.43, 0.94)	0.03	0.69* (0.43, 0.94)	0.027
Yes	Ref		Ref		Ref	
**Physical activity**						
Frequent	Ref		Ref		Ref	
Rarely	0.36* (0.13, 0.59)	0.018	0.36* (0.13, 0.59)	0.02	0.36* (0.13, 0.59)	0.018
Never	0.91* (0.73, 1.09)	0.063	0.91* (0.73, 1.09)	0.06	0.91* (0.73, 1.09)	0.063
**Ill-treated in last one year**						
Yes	− 0.01 (− 0.34, 0.32)	0.000	− 0.01 (− 0.34, 0.32)	0.00	− 0.01 (− 0.34, 0.32)	0.000
No	Ref		Ref		Ref	
**Health-related factors**						
**Depression**						
No	Ref		Ref		Ref	
Yes	0.18 (− 0.07, 0.44)	0.007	0.18 (− 0.07, 0.44)	0.01	0.18 (− 0.07, 0.44)	0.007
**Self-rated health**						
Good	Ref		Ref		Ref	
Poor	0.72* (0.59, 0.86)	0.054	0.72* (0.59, 0.86)	0.05	0.72* (0.59, 0.86)	0.054
**Difficulty in ADL**						
No	Ref		Ref		Ref	
Yes	0.66* (0.48, 0.84)	0.038	0.66* (0.48, 0.84)	0.04	0.66* (0.48, 0.84)	0.038
**Difficulty in IADL**						
No	Ref		Ref		Ref	
Yes	0.98* (0.83, 1.12)	0.072	0.98* (0.83, 1.12)	0.07	0.98* (0.83, 1.12)	0.072
**Chronic disease**						
0	Ref		Ref		Ref	
1	− 0.30* (− 0.45, − 0.14)	− 0.020	− 0.3* (− 0.45, − 0.14)	− 0.02	− 0.30* (− 0.45, − 0.14)	− 0.020
2 +	− 0.53* (− 0.70, − 0.36)	− 0.034	− 0.53* (− 0.7, − 0.36)	− 0.03	− 0.53* (− 0.70, − 0.36)	− 0.034
**Household/community factors**						
**MPCE quintile**						
Poorest	Ref		Ref		Ref	
Poorer	1.16* (0.94, 1.38)	0.068	1.16* (0.94, 1.38)	0.07	1.16* (0.94, 1.38)	0.068
Middle	0.88* (0.67, 1.09)	0.053	0.88* (0.67, 1.09)	0.05	0.88* (0.67, 1.09)	0.053
Richer	0.63* (0.43, 0.84)	0.038	0.63* (0.43, 0.83)	0.04	0.63* (0.43, 0.84)	0.038
Richest	0.44* (0.24, 0.64)	0.026	0.44* (0.24, 0.64)	0.03	0.44* (0.24, 0.64)	0.026
**Religion**						
Hindu	Ref		Ref		Ref	
Muslim	− 0.06 (− 0.27, 0.14)	− 0.003	− 0.06 (− 0.27, 0.14)	0.00	− 0.06 (− 0.27, 0.14)	− 0.003
Christian	− 0.02 (− 0.30, 0.25)	− 0.001	− 0.02 (− 0.3, 0.25)	0.00	− 0.02 (− 0.30, 0.25)	− 0.001
Others	− 0.43* (− 0.73, − 0.12)	− 0.014	− 0.43* (− 0.73, − 0.12)	− 0.01	− 0.43* (− 0.73, − 0.12)	− 0.014
**Caste**						
Scheduled Caste	Ref		Ref		Ref	
Scheduled Tribe	0.78* (0.52, 1.04)	0.041	0.79* (0.52, 1.05)	0.04	0.78* (0.52, 1.04)	0.041
Other Backward Class	− 0.59* (− 0.79, − 0.40)	− 0.043	− 0.59* (− 0.79, − 0.4)	− 0.04	− 0.59* (− 0.79, − 0.40)	− 0.043
Others	− 0.50* (− 0.71, − 0.30)	− 0.035	− 0.5* (− 0.71, − 0.3)	− 0.03	− 0.5* (− 0.71, − 0.30)	− 0.035
**Place of residence**						
Rural	Ref		Ref		Ref	
Urban	− 1.70* (− 1.85, − 1.55)	− 0.121	− 1.69* (− 1.84, − 1.54)	− 0.12	− 1.70* (− 1.85, − 1.55)	− 0.121
**Region**						
North	Ref		Ref		Ref	
Central	− 0.48* (− 0.72, − 0.25)	− 0.025	− 0.49* (− 0.72, − 0.25)	− 0.03	− 0.48* (− 0.72, − 0.25)	− 0.025
East	− 0.63* (− 0.85, − 0.41)	− 0.036	− 0.63* (− 0.85, − 0.42)	− 0.04	− 0.63* (− 0.85, − 0.41)	− 0.036
Northeast	− 0.60* (− 0.87, − 0.32)	− 0.028	− 0.6* (− 0.87, − 0.32)	− 0.03	− 0.60* (− 0.87, − 0.32)	− 0.028
West	0.34* (0.10, 0.58)	0.017	0.33* (0.1, 0.57)	0.02	0.34* (0.10, 0.58)	0.017
South	− 0.92* (− 1.13, − 0.70)	− 0.059	− 0.92* (− 1.13, − 0.71)	− 0.06	− 0.92* (− 1.13, − 0.70)	− 0.059

*if p < 0.05; #, Interaction effect; Ref, Reference; CI, Confidence interval; aCoef., Adjusted regression coefficients; Beta, Standardized beta coefficients; ADL, Activities of daily living; IADL, Instrumental activities of daily living; MPCE, Monthly per capita consumption expenditure. The analysis in model-1, model-2 and model-3 was controlled for all individual, health-related and household/community factors.

Model-2 represents the interaction estimates of childhood health and financial status on cognitive impairment among older adults. Although the actual main effects were significant, there was no statistical significance in the interaction effects. Further, Model-3 represents the stratified analysis of childhood health and childhood financial status. Older adults who had fair childhood health and poor childhood financial status were more likely to suffer from cognitive impairment in comparison to older adults who had good childhood health and good financial status (Coef: 1.26; CI 0.86, 1.66). Table S1 (supplementary file) presents the regression estimates for cognitive impairment among older adults stratified by sex along with the results of interaction and stratification of childhood factors with late-life cognitive impairment; additionally, Table S2 (supplementary file) represents the sensitivity analysis for mild cognitive impairment among older adults after excluding participants with suspected dementia by their background characteristics. Similar results were observed in this additional analysis.

## Discussion

It is fairly well established that early life childhood deprivation, environment and childhood health may contribute to cognitive impairment in the later life stages^54^. However, the risk of cognitive impairment in old age due to adverse socioeconomic conditions in childhood has been understudied in India compared to other developing countries. Such a study is particularly important in a resource-constrained setting where there is a need for wider efforts to reduce the prevalence of cognitive impairment among older adults and its burden on health care systems. This study used data from a large population-based ageing survey conducted in India, to advance understanding of childhood socioeconomic and health conditions as major factors in the early-life course that associate with cognitive function in later years of life.

As evidence suggests, children from households with higher SES may be in a more cognitively stimulating environment in their early life resulting in more advanced brain development than their disadvantaged counterparts^55^. Such advancements in the brain in the early life course are shown to be associated with better cognitive functioning in older ages^56^. Studies drawing on data from different socio-cultural settings had found that older adults, when they experienced higher levels of SES in childhood, perform better on neurocognitive tests^57^. On the other hand, it is documented that the longer people live in poor SES and health conditions, the greater would be their academic deficits and the more severe the decline in their cognitive abilities^58^. Concordantly, the present analysis provides consistent evidence that childhood SES predicts cognitive impairment in older ages. The experiences in childhood do substantially influence the health status in later life because childhood conditions predict to a great extent, the probable pathways that may lead to good or bad health. As multiple studies suggest, childhood economic resources and health determine the living and working conditions in adulthood, and those circumstances give rise to social inequalities in health^59,60^. Besides, nutritional deprivation during such important periods of early development may have negative effects on cognitive functioning in the long term^34^. Parallel to these findings, current results also suggest that compared to good childhood health, fair health condition is significantly associated with cognitive impairment among older adults. Both findings could be interpreted as evidence that childhood SES and health conditions may have a long-lasting effect on an active cognitive reserve that may have a major role in determining the rate of cognitive functioning in later years of life.

In the interaction analysis model that included a term for the interaction of childhood health and financial status, we did not find evidence for both the childhood adversities in combination increasing the significance effect on cognitive impairment. However, additional regression analysis including stratification of low childhood SES measured by a worse-off family financial status and fair childhood health conditions showed that they were statistically significant for cognitive impairment in old age. All these suggest that the effects of low childhood SES on late-life cognitive impairment were stronger for people with fair childhood health conditions than for people with good childhood health. Although fair childhood health status in the stratified estimates showed higher cognitive impairment, poor childhood health status did not show statistical significance which might be attributed to the lower sample size in the poor health category that might result in lack of statistical power in the analysis. Further longitudinal studies are warranted in developing countries like India that explore the rate of cognitive decline in old age in relation to the life course socioeconomic and health conditions. Such an investigation may further contribute to an improved understanding of the mechanisms such as lack of social and economic resources and increased illiteracy surrounding the cognitive impairment in old age and bringing interventions for early detection and prevention of cognitive impairment and related disabilities in older ages. The study also supports that the association of early life circumstances with cognitive characteristics in old age observed in high-income countries and some developing countries, including China, may extend to community-dwelling older adults in the Indian context as well.

Another particularly striking finding in our analysis was the protective effect of education on a late-life cognitive impairment that is consistent with past literature^61–63^, indicating that providing education as an intervention to diminish the adverse effects of poor childhood SES and health conditions on cognitive ageing. Significantly increased odds of lower education in relationship with cognitive impairment in our analysis support the findings of previous studies suggesting that the higher levels of education often lead to occupations that necessitate active cognitive involvements, which could further enhance or maintain cognitive functioning in late adulthood^62,64^. At the same time, children may lack the energy and motor skills essential to thrive in school due to poor household conditions and limited resources and thus complete fewer years of schooling, which in turn affects late-life cognition^65,66^. Hence, considering the findings of the present study, adverse household conditions could be an indicator for identifying the children at-risk who would benefit in the long term from targeted interventions on increasing their education.

The current findings revealed a significant female disadvantage in cognition and a stronger association of childhood health conditions with late-life cognitive functioning among women than men, where older women with a fair health status in childhood had higher odds of cognitive impairment in late-life compared to those with a good health status during childhood. This is consistent with previous studies^67^ which suggest that women are at higher disadvantage in terms of having adverse structural, behavioural and psychosocial characteristics across the lifespan that are related to poor late-life health outcomes. Again, the current finding is similar (for childhood SES) to existing studies which showed that childhood SES was associated with old-age mental health among women but not among men in models fully adjusted with adulthood SES and risk factors^68,69^. Considering the interaction results segregated by gender, having a fair childhood health and poor childhood SES had higher odds of cognitive impairment both among men and women in comparison to having a good health and SES in childhood and the odds were greater among women. On the other hand, a study based on the data from the HRS suggested that with respect to memory function, cumulative SES from childhood to adulthood may be more important among men than women^70^, which suggests the need for future studies on the influence of cumulative exposure to life-course disadvantages on late-life health with special focus on gender aspect.

There are several limitations of the present study to be acknowledged. Foremost, the cross-sectional design of the analysis in the present study prevents bringing out any causal inferences. Further, although a global measure of cognitive impairment has the advantage of assessing overall cognition, the relationship with early life health and SES is potentially different for specific domains of cognitive function. For example, memory unlike other cognitive functions is found to be more sensitive measure of age-related cognitive deficits^71,72^. Therefore, future studies on domain-specific associations are warranted. A sensitivity analysis was conducted in the current study after excluding participants who were cognitively impaired or suspected with dementias, and the results showed no changes in the observed associations. Also, measurement error in several cognitive domains may be biasing the current results due to a higher proportion of illiterate population in India (with 68% older adults with no or uncompleted primary education in this study), and thus additional research with longitudinal and interventional designs is required to unravel this issue. Notably, educational variable and its categories in the current study are equivalent to the ISCED and allow comparisons to be made with other international studies. Similarly, studies on the validity and reliability of the measurement method of cognitive impairment are recommended.

Indeed, it is also important to consider that some childhood conditions may affect cognitive functioning directly, and others may act indirectly through several pathways in adulthood^6,73^. Hence, understanding SES in adulthood as a risk/protective factor for later-life cognition is also essential for identifying the factors related to cognitive ability in older ages. Also, self-report of health conditions and SES in childhood may be subject to recall bias and information on receiving healthcare support/assistance for reported poor health conditions was not available which may bias the current results. This study, however, provides baseline data for understanding the ageing trajectories and the risk factors for cognitive impairment in late life. Further longitudinal studies with more follow-up information from upcoming waves of LASI surveys may add to this gap. Another major limitation is that given the predictor variables of interest in the study are self-reported, there are greater chances of recall bias, especially in the case of childhood conditions. However, the study has the credit of utilizing the large survey information of the older population, which is nationally representative and provides comprehensive measures of cognitive functioning in an ageing population.

## Conclusions

The current study's findings highlight the necessity of determining whether certain developmental periods are linked to cognitive impairment later in life. Our findings also imply that governments should place a greater emphasis on closing socioeconomic resource inequalities across the lifespan, especially in childhood. Furthermore, there are various windows of opportunity for age-based interventions, with those in the early years of life shaping individuals' socioeconomic paths into later life being the most promising. As a result, social measures such as increasing educational and financial resources in disadvantaged neighbourhoods and socioeconomically poor children and their families may aid in cognitive ageing and a healthy and dignified life in old age. Without a question, socioeconomic measures aimed at improving childhood conditions are critical, as here is where an incremental route to long-term physical as well as mental health begins.

## Supplementary Information

Supplementary Information.

## Data Availability

The datasets generated and/or analysed during the current study are available in the International Institute for Population Institute’s repository, [https://iipsindia.ac.in/sites/default/files/LASI_DataRequestForm_0.pdf].

## Abbreviations

IADL: Instrumental activities of daily living
ADL: Activities of daily living
MPCE: Monthly per capita consumption expenditure
SES: Socio-economic status
LASI: Longitudinal Ageing Study in India
CIDI-SF: Short Form Composite International Diagnostic Interview
