# Supplementary material for: Childhood deprivations predict late-life cognitive impairment among older adults in India
Source: Sci Rep. 2022 Jul 27;12:12786. doi: 10.1038/s41598-022-16652-y (PMC9329336; doi:10.1038/s41598-022-16652-y)
Supplement: Supplementary file 1 — Supplementary Information. [file 41598_2022_16652_MOESM1_ESM.pdf]

Table-S1. Regression estimates for cognitive impairment among older adults stratified by sex

Background Factors	Male						Female					
	Model-1		Model-2		Model-3		Model-1		Model-2		Model-3	
	aCoef. (95% CI)	Beta	aCoef. (95% CI)	Beta	aCoef. (95% CI)	Beta	aCoef. (95% CI)	Beta	aCoef. (95% CI)	Beta	aCoef. (95% CI)	Beta
**Childhood deprivation factors**												
**Childhood health status**												
Good	Ref.		Ref.				Ref.		Ref.			
Fair	0.5*(0.21,0.8)	0.024	0.32(-1.21,1.84)	0.015			0.62*(0.32,0.92)	0.029	0.64(-0.69,1.97)	0.030		
Poor	-0.36(-1.05,0.34)	-0.007	1.43(-2,4.86)	0.029			-0.23(-0.97,0.51)	-0.004	0.17(-2.5,2.84)	0.003		
**Childhood financial status**												
Good	Ref.		Ref.				Ref.		Ref.			
Average	0.11(-0.23,0.46)	0.009	0.1(-0.25,0.45)	0.008			0.62*(0.32,0.92)	-0.003	-0.03(-0.38,0.32)	-0.002		
Poor	0.56*(0.2,0.92)	0.045	0.6*(0.23,0.97)	0.048			-0.23(-0.97,0.51)	0.068	0.93*(0.56,1.31)	0.068		
**Childhood health status # Childhood financial status**												
Fair # Average			0.38(-1.19,1.96)	0.014					0.02(-1.37,1.4)	0.001		
Fair # Poor			-0.09(-1.69,1.51)	-0.003					-0.09(-1.5,1.33)	-0.003		
Poor # Average			-1.51(-5.13,2.1)	-0.019					-0.68(-3.58,2.22)	-0.008		
Poor # Poor			-2.1(-5.64,1.45)	-0.033					-0.24(-3.1,2.62)	-0.003		
**Status of childhood health & Financial status of family during childhood**												
Good & good					Ref.						Ref.	
Good & average					0.1(-0.25,0.45)	0.008					-0.03(-0.38,0.32)	-0.002
Good & poor					0.6*(0.23,0.97)	0.047					0.93*(0.56,1.31)	0.066
Fair & good					0.32(-1.21,1.84)	0.003					0.64(-0.69,1.97)	0.007
Fair & average					0.8*(0.31,1.29)	0.03					0.63*(0.14,1.12)	0.023
Fair & poor					0.83*(0.26,1.39)	0.025					1.49*(0.92,2.05)	0.044
Poor & good					1.43(-2,4.86)	0.006					0.17(-2.5,2.84)	0.001
Poor & average					0.02(-1.17,1.2)	0.000					-0.54(-1.72,0.64)	-0.006
Poor & poor					-0.06(-1.02,0.89)	-0.001					0.86(-0.22,1.95)	0.011
**Individual factors**												
**Age**												
Young-old	Ref.		Ref.		Ref.		Ref.		Ref.		Ref.	
Old-old	0.73*(0.53,0.93)	0.054	0.73*(0.53,0.93)	0.054	0.73*(0.53,0.93)	0.054	1.33*(1.11,1.55)	0.089	1.33*(1.11,1.55)	0.089	1.33*(1.11,1.55)	0.089
Oldest-old	2.33*(2,2.67)	0.108	2.33*(2,2.67)	0.108	2.33*(2,2.67)	0.108	2.97*(2.61,3.33)	0.123	2.97*(2.61,3.33)	0.123	2.97*(2.61,3.33)	0.123
**Education**												
No education/primary not completed	6.38*(6.05,6.71)	0.518	6.38*(6.05,6.71)	0.518	6.38*(6.05,6.71)	0.518	8.41*(7.9,8.92)	0.547	8.41*(7.9,8.92)	0.547	8.41*(7.9,8.92)	0.547
Primary	2.73*(2.38,3.08)	0.162	2.73*(2.38,3.08)	0.162	2.73*(2.38,3.08)	0.162	3.67*(3.12,4.22)	0.167	3.67*(3.12,4.22)	0.167	3.67*(3.12,4.22)	0.167
Secondary	1.19*(0.88,1.49)	0.082	1.19*(0.88,1.49)	0.082	1.19*(0.88,1.49)	0.082	1.82*(1.29,2.36)	0.082	1.82*(1.29,2.36)	0.08	1.82*(1.29,2.36)	0.082
Higher	Ref.		Ref.		Ref.		Ref.		Ref.		Ref.	
**Working status**												
Never worked	0.5*(0.06,0.93)	0.017	0.5*(0.06,0.93)	0.017	0.5*(0.06,0.93)	0.017	0.54*(0.26,0.83)	0.041	0.54*(0.26,0.83)	0.041	0.54*(0.26,0.83)	0.041
Currently working	Ref.		Ref.		Ref.		Ref.		Ref.		Ref.	
Not currently working	0.09(-0.14,0.31)	0.007	0.09(-0.14,0.31)	0.007	0.09(-0.14,0.31)	0.007	0.24(-0.05,0.53)	0.017	0.24(-0.05,0.53)	0.017	0.24(-0.05,0.53)	0.017
Retired	-0.57*(-0.86,-0.29)	-0.035	-0.57*(-0.86,-0.29)	-0.035	-0.57*(-0.86,-0.29)	-0.035	-1.36*(-2.01,-0.72)	-0.033	-1.36*(-2.01,-0.72)	-0.033	-1.36*(-2.01,-0.72)	-0.033
**Marital status**												
Currently married	Ref.		Ref.		Ref.		Ref.		Ref.		Ref.	
Widowed	0.6*(0.32,0.89)	0.034	0.6*(0.32,0.89)	0.034	0.6*(0.32,0.89)	0.034	0.75*(0.52,0.98)	0.057	0.75*(0.52,0.98)	0.057	0.75*(0.52,0.98)	0.057

Others	0.1(-0.55,0.76)	0.003	0.1(-0.55,0.76)	0.003	0.1(-0.55,0.76)	0.003	0.02(-0.6,0.64)	0	0.02(-0.6,0.64)	0.000	0.02(-0.6,0.64)	0
**Living arrangement**												
Living alone	Ref.		Ref.		Ref.		Ref.		Ref.		Ref.	
Living with spouse	0.48(-0.16,1.12)	0.033	0.48(-0.16,1.12)	0.033	0.48(-0.16,1.12)	0.033	0.2(-0.25,0.66)	0.011	0.2(-0.25,0.66)	0.011	0.2(-0.25,0.66)	0.011
Living with children and spouse	0.19(-0.42,0.8)	0.014	0.19(-0.42,0.8)	0.014	0.19(-0.42,0.8)	0.014	-0.27(-0.64,0.11)	-0.018	-0.27(-0.64,0.11)	-0.018	-0.27(-0.64,0.11)	-0.018
Living with others.	0.79*(0.03,1.55)	0.023	0.79*(0.03,1.55)	0.023	0.79*(0.03,1.55)	0.023	0.31(-0.19,0.8)	0.011	0.31(-0.19,0.8)	0.011	0.31(-0.19,0.8)	0.011
**Social participation**												
No	0.68*(0.37,1)	0.032	0.68*(0.37,1)	0.032	0.68*(0.37,1)	0.032	0.85*(0.44,1.26)	0.03	0.85*(0.44,1.26)	0.030	0.85*(0.44,1.26)	0.03
Yes	Ref.		Ref.		Ref.		Ref.		Ref.		Ref.	
**Physical activity**												
Frequent	Ref.		Ref.		Ref.		Ref.		Ref.		Ref.	
Rarely	0.31*(0.03,0.59)	0.018	0.31*(0.03,0.59)	0.018	0.31*(0.03,0.59)	0.018	0.43*(0.06,0.81)	0.02	0.43*(0.06,0.81)	0.020	0.43*(0.06,0.81)	0.02
Never	0.8*(0.57,1.03)	0.064	0.8*(0.57,1.03)	0.064	0.8*(0.57,1.03)	0.064	1.03*(0.73,1.32)	0.066	1.03*(0.73,1.32)	0.066	1.03*(0.73,1.32)	0.066
**Ill-treated in last one year***												
Yes	0.08(-0.39,0.55)	0.002	0.08(-0.39,0.55)	0.002	0.08(-0.39,0.55)	0.002	-0.14(-0.59,0.32)	-0.004	-0.14(-0.59,0.32)	-0.004	-0.14(-0.59,0.32)	-0.004
No	Ref.		Ref.		Ref.		Ref.		Ref.		Ref.	
**Health factors**												
**Depression***												
No	Ref.		Ref.		Ref.		Ref.		Ref.		Ref.	
Yes	0.07(-0.3,0.43)	0.003	0.07(-0.3,0.43)	0.003	0.07(-0.3,0.43)	0.003	0.24(-0.12,0.59)	0.009	0.24(-0.12,0.59)	0.009	0.24(-0.12,0.59)	0.009
**Self-rated health***												
Good	Ref.				Ref.		Ref.		Ref.		Ref.	
Poor	0.71*(0.52,0.9)	0.057	0.71*(0.52,0.9)	0.057	0.71*(0.52,0.9)	0.057	0.78*(0.58,0.97)	0.059	0.78*(0.58,0.97)	0.059	0.78*(0.58,0.97)	0.059
**Difficulty in ADL***												
No	Ref.				Ref.		Ref.		Ref.		Ref.	
Yes	0.66*(0.39,0.92)	0.039	0.66*(0.39,0.92)	0.039	0.66*(0.39,0.92)	0.039	0.67*(0.43,0.92)	0.042	0.67*(0.43,0.92)	0.042	0.67*(0.43,0.92)	0.042
**Difficulty in IADL***												
No	Ref.		Ref.		Ref.		Ref.		Ref.		Ref.	
Yes	1.1*(0.88,1.31)	0.083	1.1*(0.88,1.31)	0.083	1.1*(0.88,1.31)	0.083	0.81*(0.61,1.02)	0.061	0.81*(0.61,1.02)	0.061	0.81*(0.61,1.02)	0.061
**Chronic disease**												
0	Ref.		Ref.		Ref.		Ref.		Ref.		Ref.	
1	-0.24*(-0.45,-0.03)	-0.018	-0.24*(-0.45,-0.03)	-0.018	-0.24*(-0.45,-0.03)	-0.018	-0.39*(-0.61,-0.17)	-0.027	-0.39*(-0.61,-0.17)	-0.027 -0.049	-0.39*(-0.61,-0.17)	-0.027
2+	-0.3*(-0.54,-0.06)	-0.021	-0.3*(-0.54,-0.06)	-0.021	-0.3*(-0.54,-0.06)	-0.021	-0.74*(-0.99,-0.49)	-0.049	-0.74*(-0.99,-0.49)		-0.74*(-0.99,-0.49)	-0.049
**Household factors**												
**MPCE quintile**												
Poorest	Ref.		Ref.		Ref.		Ref.		Ref.		Ref.	
Poorer	0.92*(0.62,1.22)	0.058	0.92*(0.62,1.22)	0.058	0.92*(0.62,1.22)	0.058	1.34*(1.03,1.66)	0.081	1.34*(1.03,1.66)	0.081	1.34*(1.03,1.66)	0.081
Middle	0.83*(0.54,1.12)	0.054	0.83*(0.54,1.12)	0.054	0.83*(0.54,1.12)	0.054	0.9*(0.6,1.2)	0.055	0.9*(0.6,1.2)	0.055	0.9*(0.6,1.2)	0.055
Richer	0.45*(0.17,0.73)	0.03	0.45*(0.17,0.73)	0.030	0.45*(0.17,0.73)	0.03	0.78*(0.49,1.08)	0.048	0.78*(0.49,1.08)	0.048	0.78*(0.49,1.08)	0.048
Richest	0.44*(0.17,0.72)	0.029	0.44*(0.17,0.72)	0.029	0.44*(0.17,0.72)	0.029	0.43*(0.14,0.72)	0.026	0.43*(0.14,0.72)	0.026	0.43*(0.14,0.72)	0.026
**Religion**												
Hindu	Ref.		Ref.		Ref.		Ref.		Ref.		Ref.	
Muslim	-0.71*(-0.99,-0.43)	-0.038	-0.71*(-0.99,-0.43)	-0.038	-0.71*(-0.99,-0.43)	-0.038	0.61*(0.31,0.91)	0.029	0.61*(0.31,0.91)	0.029	0.61*(0.31,0.91)	0.029
Christian	0.42*(0.04,0.8)	0.02	0.42*(0.04,0.8)	0.020	0.42*(0.04,0.8)	0.02	-0.3(-0.69,0.09)	-0.013	-0.3(-0.69,0.09)	-0.013	-0.3(-0.69,0.09)	-0.013
Others	0.01(-0.41,0.43)	0	0.01(-0.41,0.43)	0.000	0.01(-0.41,0.43)	0	-0.78*(-1.21,-0.35)	-0.026	-0.78*(-1.21,-0.35)	-0.026	-0.78*(-1.21,-0.35)	-0.026
**Caste**												
Scheduled Caste	Ref.		Ref.		Ref.		Ref.		Ref.		Ref.	
Scheduled Tribe	0.81*(0.45,1.17)	0.046	0.81*(0.45,1.17)	0.046	0.81*(0.45,1.17)	0.046	0.76*(0.38,1.14)	0.04	0.76*(0.38,1.14)	0.040	0.76*(0.38,1.14)	0.04
Other Backward Class	-0.45*(-0.72,-0.19)	-0.036	-0.45*(-0.72,-0.19)	-0.036	-0.45*(-0.72,-0.19)	-0.036	-0.76*(-1.03,-0.48)	-0.056	-0.76*(-1.03,-0.48)	-0.056	-0.76*(-1.03,-0.48)	-0.056

Others	-0.25(-0.53,0.04)	-0.018	-0.25(-0.53,0.04)	-0.018	-0.25(-0.53,0.04)	-0.018	-0.73*(-1.03,-0.44)	-0.052	-0.73*(-1.03,-0.44)	-0.052	-0.73*(-1.03,-0.44)	-0.052
**Place of residence**												
Rural	Ref.		Ref.		Ref.		Ref.		Ref.		Ref.	
Urban	-1.35*(-1.55,-1.14)	-0.104	-1.35*(-1.55,-1.14)	-0.104	-1.35*(-1.55,-1.14)	-0.104	-1.89*(-2.11,-1.68)	-0.139	-1.89*(-2.11,-1.68)	-0.139	-1.89*(-2.11,-1.68)	-0.139
**Region**												
North	Ref.		Ref.		Ref.		Ref.		Ref.		Ref.	
Central	-0.4*(-0.72,-0.08)	-0.023	-0.4*(-0.72,-0.08)	-0.023	-0.4*(-0.72,-0.08)	-0.023	-0.57*(-0.91,-0.24)	-0.03	-0.57*(-0.91,-0.24)	-0.030	-0.57*(-0.91,-0.24)	-0.03
East	-0.45*(-0.75,-0.15)	-0.029	-0.45*(-0.75,-0.15)	-0.029	-0.45*(-0.75,-0.15)	-0.029	-0.8*(-1.12,-0.48)	-0.046	-0.8*(-1.12,-0.48)	-0.046	-0.8*(-1.12,-0.48)	-0.046
Northeast	-0.32(-0.7,0.06)	-0.017	-0.32(-0.7,0.06)	-0.017	-0.32(-0.7,0.06)	-0.017	-0.92*(-1.32,-0.51)	-0.044	-0.92*(-1.32,-0.51)	-0.044	-0.92*(-1.32,-0.51)	-0.044
West	0.49*(0.16,0.83)	0.026	0.49*(0.16,0.83)	0.026	0.49*(0.16,0.83)	0.026	0.22(-0.12,0.56)	0.011	0.22(-0.12,0.56)	0.011	0.22(-0.12,0.56)	0.011
South	-0.45*(-0.74,-0.15)	-0.031	-0.45*(-0.74,-0.15)	-0.031	-0.45*(-0.74,-0.15)	-0.031	-1.32*(-1.63,-1.01)	-0.086	-1.32*(-1.63,-1.01)	-0.09	-1.32*(-1.63,-1.01)	-0.086

*if p<0.05; #: Interaction effect; Ref: Reference; CI: Confidence interval; aCoef: Adjusted Coefficients; ADL: Activities of Daily Living; IADL: Instrumental Activities of Daily Living.

**Table-S2.** Sensitivity analysis for cognitive impairment among older adults by their background characteristics in India						
**Background Factors**	**Model-1**		**Model-2**		**Model-3**	
	**aCoef. (95% CI)**	**Beta**	**aCoef. (95% CI)**	**Beta**	**aCoef. (95% CI)**	**Beta**
**Childhood deprivation factors**						
**Childhood health status**						
Good	Ref.		Ref.			
Fair	0.51*(0.31,0.71)	0.027	0.88(-0.02,1.79)	0.047		
Poor	-0.41(-0.9,0.08)	-0.009	0.75(-1.22,2.71)	0.016		
**Childhood financial status**						
Good	Ref.		Ref.			
Average	-0.01(-0.23,0.21)	-0.001	0(-0.23,0.22)	0.000		
Poor	0.5*(0.26,0.73)	0.043	0.56*(0.32,0.81)	0.049		
**Childhood health status # Childhood financial status**						
Fair # Average			-0.22(-1.16,0.72)	-0.009		
Fair # Poor			-0.67(-1.63,0.3)	-0.022		
Poor # Average			-1.01(-3.11,1.1)	-0.014		
Poor # Poor			-1.33(-3.41,0.74)	-0.021		
**Status of childhood health & Financial status of a family during childhood**						
Good & good					Ref.	
Good & average					0(-0.23,0.22)	0.000
Good & poor					0.56*(0.32,0.81)	0.048
Fair & good					0.88(-0.02,1.79)	0.010
Fair & average					0.66*(0.34,0.98)	0.028
Fair & poor					0.78*(0.4,1.16)	0.026
Poor & good					0.75(-1.22,2.71)	0.004
Poor & average					-0.26(-1.03,0.51)	-0.004
Poor & poor					-0.02(-0.7,0.66)	0.000
**Individual factors**						
**Age**						
Young-old	Ref.		Ref.		Ref.	
Old-old	0.71*(0.57,0.85)	0.057	0.71*(0.57,0.85)	0.057	0.71*(0.57,0.85)	0.057
Oldest-old	1.42*(1.17,1.66)	0.065	1.41*(1.17,1.66)	0.065	1.42*(1.17,1.66)	0.065
**Sex**						
Male	Ref.		Ref.		Ref.	
Female	1.19*(1.04,1.35)	0.107	1.19*(1.03,1.34)	0.106	1.19*(1.04,1.35)	0.107
**Education**						
No education/primary not completed	6.28*(6.04,6.52)	0.560	6.28*(6.04,6.52)	0.560	6.28*(6.04,6.52)	0.560
Primary	3.01*(2.75,3.27)	0.190	3.01*(2.75,3.27)	0.190	3.01*(2.75,3.27)	0.190
Secondary	1.41*(1.18,1.65)	0.100	1.42*(1.18,1.65)	0.100	1.41*(1.18,1.65)	0.100
Higher	Ref.		Ref.		Ref.	
**Marital status**						
Currently married	Ref.		Ref.		Ref.	
Widowed	0.4*(0.23,0.56)	0.032	0.4*(0.23,0.56)	0.032	0.4*(0.23,0.56)	0.032
Others	-0.29(-0.7,0.12)	-0.008	-0.31(-0.72,0.11)	-0.009	-0.29(-0.7,0.12)	-0.008
**Living arrangement**						
Living alone	Ref.		Ref.		Ref.	

Living with spouse	0.09(-0.25,0.43)	0.007	0.09(-0.25,0.42)	0.006	0.09(-0.25,0.43)	0.007
Living with children and spouse	-0.18(-0.49,0.12)	-0.015	-0.18(-0.49,0.12)	-0.015	-0.18(-0.49,0.12)	-0.015
Living with others.	0.13(-0.27,0.54)	0.005	0.14(-0.26,0.54)	0.005	0.13(-0.27,0.54)	0.005
**Working status**						
Never worked	0.17(-0.02,0.37)	0.014	0.17(-0.02,0.37)	0.014	0.17(-0.02,0.37)	0.014
Currently working	Ref.		Ref.		Ref.	
Not currently working	0.02(-0.14,0.18)	0.002	0.02(-0.14,0.18)	0.002	0.02(-0.14,0.18)	0.002
Retired	-0.48*(-0.71,-0.25)	-0.027	-0.48*(-0.71,-0.25)	-0.027	-0.48*(-0.71,-0.25)	-0.027
**Social participation**						
No	0.39*(0.16,0.61)	0.019	0.39*(0.16,0.61)	0.019	0.39*(0.16,0.61)	0.019
Yes	Ref.		Ref.		Ref.	
**Physical activity**						
Frequent	Ref.		Ref.		Ref.	
Rarely	0.35*(0.14,0.55)	0.021	0.34*(0.14,0.55)	0.021	0.35*(0.14,0.55)	0.021
Never	0.74*(0.58,0.9)	0.063	0.74*(0.58,0.9)	0.063	0.74*(0.58,0.9)	0.063
**Ill-treated in last one year**						
Yes	0.03(-0.28,0.34)	0.001	0.03(-0.27,0.34)	0.001	0.03(-0.28,0.34)	0.001
No	Ref.		Ref.		Ref.	
**Health factors**						
**Depression**						
No	Ref.		Ref.		Ref.	
Yes	0.04(-0.2,0.28)	0.002	0.04(-0.2,0.28)	0.002	0.04(-0.2,0.28)	0.002
**Self-rated health**						
Good	Ref.		Ref.		Ref.	
Poor	0.55*(0.42,0.68)	0.049	0.55*(0.42,0.67)	0.049	0.55*(0.42,0.68)	0.049
**Difficulty in ADL**						
No	Ref.		Ref.		Ref.	
Yes	0.25*(0.08,0.42)	0.017	0.25*(0.08,0.42)	0.017	0.25*(0.08,0.42)	0.017
**Difficulty in IADL**						
No	Ref.		Ref.		Ref.	
Yes	0.79*(0.65,0.93)	0.069	0.79*(0.65,0.92)	0.069	0.79*(0.65,0.93)	0.069
**Chronic disease**						
0	Ref.		Ref.		Ref.	
1	-0.13(-0.27,0.02)	-0.010	-0.13(-0.27,0.01)	-0.011	-0.13(-0.27,0.02)	-0.010
2+	-0.28*(-0.44,-0.12)	-0.022	-0.28*(-0.44,-0.12)	-0.022	-0.28*(-0.44,-0.12)	-0.022
**Household factors**						
**MPCE quintile**						
Poorest	Ref.		Ref.		Ref.	
Poorer	0.87*(0.67,1.08)	0.061	0.87*(0.67,1.08)	0.061	0.87*(0.67,1.08)	0.061
Middle	0.74*(0.55,0.93)	0.053	0.74*(0.54,0.93)	0.053	0.74*(0.55,0.93)	0.053
Richer	0.54*(0.36,0.73)	0.040	0.54*(0.36,0.73)	0.040	0.54*(0.36,0.73)	0.040
Richest	0.42*(0.23,0.6)	0.030	0.42*(0.23,0.6)	0.030	0.42*(0.23,0.6)	0.030
**Religion**						
Hindu	Ref.		Ref.		Ref.	
Muslim	-0.07(-0.26,0.12)	-0.004	-0.07(-0.26,0.12)	-0.004	-0.07(-0.26,0.12)	-0.004
Christian	0.11(-0.15,0.36)	0.006	0.11(-0.15,0.36)	0.005	0.11(-0.15,0.36)	0.006

Others	-0.29*(-0.57,-0.01)	-0.011	-0.29*(-0.57,-0.01)	-0.011	-0.29*(-0.57,-0.01)	-0.011
**Caste**						
Scheduled Caste	Ref.		Ref.		Ref.	
Scheduled Tribe	0.41*(0.16,0.66)	0.025	0.42*(0.17,0.67)	0.026	0.41*(0.16,0.66)	0.025
Other Backward Class	-0.42*(-0.6,-0.24)	-0.037	-0.42*(-0.6,-0.24)	-0.037	-0.42*(-0.6,-0.24)	-0.037
Others	-0.36*(-0.55,-0.17)	-0.030	-0.36*(-0.55,-0.17)	-0.030	-0.36*(-0.55,-0.17)	-0.030
**Place of residence**						
Rural	Ref.		Ref.		Ref.	
Urban	-1.31*(-1.44,-1.17)	-0.114	-1.31*(-1.44,-1.17)	-0.114	-1.31*(-1.44,-1.17)	-0.114
**Region**						
North	Ref.		Ref.		Ref.	
Central	-0.31*(-0.52,-0.09)	-0.019	-0.31*(-0.52,-0.09)	-0.019	-0.31*(-0.52,-0.09)	-0.019
East	-0.53*(-0.73,-0.32)	-0.036	-0.53*(-0.73,-0.33)	-0.037	-0.53*(-0.73,-0.32)	-0.036
Northeast	-0.76*(-1.02,-0.5)	-0.043	-0.76*(-1.02,-0.5)	-0.043	-0.76*(-1.02,-0.5)	-0.043
West	0.25*(0.03,0.47)	0.015	0.25*(0.03,0.47)	0.015	0.25*(0.03,0.47)	0.015
South	-0.93*(-1.13,-0.73)	-0.072	-0.93*(-1.13,-0.73)	-0.072	-0.93*(-1.13,-0.73)	-0.072

*if p<0.05; #: Interaction effect; Ref: Reference; CI: Confidence interval; aCoef: Adjusted Coefficients; ADL: Activities of Daily Living; IADL: Instrumental Activities of Daily Living.
